# Gut Biogeography Accentuates Sex-Related Differences in the Murine Microbiome

**DOI:** 10.3390/microorganisms12010221

**Published:** 2024-01-22

**Authors:** Melanie Ortiz-Alvarez de la Campa, Noelle Curtis-Joseph, Chapman Beekman, Peter Belenky

**Affiliations:** 1 Department of Molecular Microbiology and Immunology, Brown University, Providence, RI 02912, USA; 2 Department of Molecular Biology, Cellular Biology, and Biochemistry, Brown University, Providence, RI 02912, USA

**Keywords:** gut microbiota, upper gastrointestinal tract, sex differences, murine microbiome

## Abstract

Recent studies have highlighted the influence of factors such as sex and sex-linked hormones on microbiome composition, raising concerns about the generalizability of findings. Here, we explore whether gut geography, specifically the upper and lower gastrointestinal tract (GI), contributes to sex-linked microbiome differences in mice. We collected microbial samples throughout the length of the GI from male and female C57B6/J mice at 6- and 8-weeks old, and conducted 16S rRNA sequencing. Our findings revealed significant sex-related differences, with *Clostridium_sensu_stricto_1* more abundant in the male colon, while females exhibited higher levels of *Dubosiella newyorkensis* across all organs at 6 weeks. We also observed decreased Shannon alpha diversity in the small intestine compared to the lower GI, and this diversity decreased further at 8 weeks. Interestingly, our results suggest that age mitigates sex-related, but not gut geography-related differences in beta diversity, with implications for experimental outcomes and treatment strategies. This study underscores the dynamic nature of microbial diversity, influenced by sex, age, and GI localization, emphasizing the need for a more comprehensive understanding of microbiome dynamics in experimental research and clinical interventions.

## 1. Introduction

The human microbiome is the term used to describe the collection of all microorganisms in the GI tract, as well as their spectrum of activity [1]. This complex microbial community has been shown to be impacted by a number of intrinsic and extrinsic factors. Extrinsic factors include diet, pharmaceutical use, and pollution, to name a few [2,3,4]. Similarly, intrinsic factors such as age [5], immunity [6], and body site [7] can also have significant impacts on the microbiome, and associations to conditions such as inflammatory bowel disease (IBD) [8], allergies [9], and autoimmune disorders [10].

The microbiota has also been shown to vary between females and males [11]. More recently, the field has evaluated potential drivers of sex differences in the microbiome, such as the interplay between sex hormones, immune responses, and genetic factors [12]. Estrogen has been associated with increased microbial diversity and altered community structure [13]. Testosterone, on the other hand, has been linked to changes in skin permeability and metabolism, which can indirectly influence the microbiome [14,15]. Progesterone, known for its role in growth and development, has also been linked to microbial composition changes [16]. In males, progesterone contributes to testosterone formation and spermatogenesis; in females, it increases the urge to mate in the estrous cycle. All three hormones can be found in varying quantities in both sexes.

However, it is important to note that most investigations exploring sex differences in the microbiome have primarily focused on the lower GI and in particular on the fecal material. The upper GI, including the stomach and small intestine, exhibits distinct physiological and environmental characteristics compared to the lower GI [17]. Previous studies have also identified significant diversity and compositional differences between the lower and the upper GI when it comes to microbiome samples [18]. These regional differences may introduce additional complexities and contribute to sex-related variations in the microbiome. This research gap warrants further exploration to understand if such sex-related microbiome differences are observed across the entire GI tract. Therefore, we chose to examine both the upper and lower GI microbiome at two timepoints to determine if sex-related microbiome variations were shaped by gut geography, sexual maturity, and/or hormone levels.

## 2. Materials and Methods

### 2.1. Mice and Sample Collection

All animal work was conducted in compliance with the protocols approved by the Institutional Animal Care and Use Committee (IACUC) of Brown University. A total of thirty-two (32) female and male C57BL/6J mice were obtained from Jackson Laboratories (Bar Harbor, ME, USA) at 4 weeks old and allowed to acclimate to our facility. Mice were sacrificed at two timepoints: 6 weeks of age and 8 weeks of age. In total, 19 and 13 mice were sacrificed at the 6-week timepoint and the 8-week timepoint, respectively. All experiments were carried out with replicates spanning multiple experimental cohorts. Microbial samples were collected from sections of the entire GI tract by squeezing the internal contents of the tissue into a 1.5 mL Eppendorf tube and excluding host tissue. Samples were immediately placed on ice <60 min and then stored at −80 °C until extraction. The GI tract was separated into the following categories: upper GI (stomach, proximal small intestine, middle small intestine, and distal small intestine) and lower GI (cecum and colon). The small intestine was defined as the end of the stomach until the beginning of the cecum, and then cut into three same length sections named in order from stomach–cecum: proximal, middle, and distal. Whole blood was also collected via cardiac puncture.

### 2.2. Microbial Sample Preparation and Analysis

DNA was extracted from the GI contents using the ZymoBiomics DNA Miniprep Kit (Zymo Research, Irvine, CA, USA, Cat #: D4300). The samples were barcoded using the Earth Microbiome Project 806R and 515F primers [19,20], and PCR amplicons targeting the bacterial 16S rRNA gene (V4 hypervariable region) were created using Phusion High-Fidelity Polymerase (New England Biolabs, Ipswich, MA, USA). The PCR conditions were as follows: denaturing at 98 °C for 3 min 45 s, annealing at 50 °C for 1 min, extension at 72 °C for 1 min 30 s, 35 cycles, and a final extension at 72 °C for 10 min.

Samples were pooled into a single library and cleaned using the NucleoSpin Gel and PCR Clean-up Kit (Macharey-Nagel, Düren, Germany, Item #:740609.50). The prepared library was sent to the Rhode Island Genomics and Sequencing Center at the University of Rhode Island (Kingston, RI, USA) for quality control and sequencing. Samples were paired-end sequenced (2 × 250 bp) using the 500-cycle kit standard protocols on an Illumina MiSeq platform.

The raw paired-end FASTQ reads were processed using the Quantitative Insights Into Microbial Ecology 2 pipeline (QIIME2, ver. 2021.11, https://qiime2.org/, accessed on 2 December 2023). Quality reads were filtered, trimmed, and denoised using the Divisive Amplicon Denoising Algorithm 2 (DADA2). Samples with less than 1000 reads were not considered in the analysis. A taxonomic assignment was performed using the SILVA database through the QIIME2 pipeline. Less than 1% of filtered reads were unassigned. Subsequent relative abundance counts, as well as alpha and beta diversity metrics, were obtained using the R (v.4.2.2) package phyloseq [21].

### 2.3. Blood Serum Preparation and Analysis

Whole blood collected from the mice was allowed to clot and separate at room temperature for 30 min in a DNA Lo-Bind Eppendorf 1.5 mL tube. Then, the blood samples were centrifuged at 2000× *g* for 10 min at 4 °C. The supernatant (serum) was transferred into a new tube and placed on ice. Serum was stored in 0.5 mL aliquots at −20 °C until analysis. Hormone concentrations in the serum were quantified via ELISA using the following kits: Mouse Testosterone ELISA Kit (Crystal Chem, Elk Grove Village, IL, USA, Cat #: 80552), Rat Estradiol ELISA Kit (Crystal Chem, Cat #: 80548), and Mouse Progesterone ELISA Kit (Crystal Chem, Cat #: 80559). All kits were performed according to the manufacturer’s instructions.

### 2.4. Bioinformatic Analysis

Differential abundance analysis was conducted using the DESeq2 R package [22] (v.3.18). Linear discriminant analysis effect size was performed using the LEfSe plug-in provided by the Galaxy server of the Huttenhower lab (Galaxy Version 1.39.5.0, https://usegalaxy.org/, accessed on 25 May 2023). Multivariable associations were calculated using the Microbiome Multivariable Associations with Linear Models (MaAsLin2) R package (v.1.4.0) [23]. Permutational multivariate analysis of variance (PERMANOVA) was conducted on the beta diversity distance matrix output from QIIME2 (v.2022.2) using the adonis2 function in the R package “vegan” (v.2.6-4) [24].

## 3. Results and Discussion

**Figure 1:**
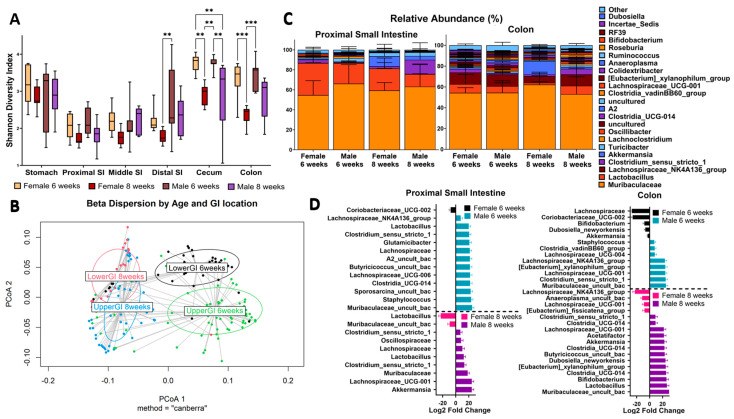
Sex and Age Differences Vary Between Upper and Lower GI. (**A**) Shannon alpha diversity throughout all collected GI locations for both sexes at 6 and 8 weeks old. Significance values as follows: ** *p* < 0.01, *** *p* < 0.001. Non-significant values are not labeled. (**B**) Beta dispersion analysis using Canberra method comparing upper and lower GI samples at 6 and 8 weeks old (*p*-val: 0.029). (**C**) Genus-level relative abundance plot of proximal small intestine and colon for both sexes at 6 and 8 weeks old. “Other” label refers to the sum of the rest of the features found in each group (beyond the top 23). “Uncultured” is a SILVA database term which refers to samples for which the genus-level taxonomic assignment was resolved, but this level was unassigned/unannotated in the SILVA database itself. Repeated labels correspond to different OTUs. (**D**) Taxa significantly associated with sex and proximal small intestine or colon calculated with DESeq2 R package (v.1.40.2).

As a global view, we initially looked at measures of alpha and beta diversity across all the OTUs that were identified. We utilized the Shannon index to examine the alpha diversity in the stomach, PSI, MSI, DSI, cecum, and colon of age 6- and 8-week-old male and female mice (Figure 1A). Compared to the lower GI, we observed a large decrease in diversity, generally, in the small intestine. This is consistent with the previous findings, showing that these organs are low in bacterial abundance and diversity [25]. Surprisingly, we observed a much higher alpha diversity in the stomach compared to the rest of the upper GI (PSI). This result may be an indication of coprophagy and possible amplification of bacterial DNA ingested from the lower GI. At 6 weeks of age, female and male mice have a relatively similar alpha diversity index. We found a statistically significant decrease in diversity in both females and males in the lower GI at 8 weeks of age compared to 6 weeks (Figure 1A). Interestingly, the diversity in these samples seemed to unilaterally decrease as the mice reached sexual maturity, as shown in the significant decrease between 6 and 8 weeks of age. This suggests that as the mice aged and reached the peak of sexual maturity, there was a stabilization of the microbial community in their gut. 

Our beta diversity analysis found that the unweighted UniFrac diversity was significantly different between GI location, sex, and age (*p*-val: 0.001 ***). Yet, the weighted UniFrac results were not significant, which suggested that the changes between these factors are likely found within low-abundance taxa. To confirm this, we then conducted a permutational multivariate analysis of variance (PERMANOVA) on the Bray–Curtis dissimilarity matrix. We chose the Bray–Curtis method because it is weighted by the abundance of operational taxonomic units (OTUs) without factoring phylogeny. If significant, this would suggest that the differences we observed were not limited to low-abundance taxa. We found that the upper and lower GI communities were significantly different from each other within each timepoint (6 weeks upper vs. lower GI [0.000999 ***]; 8 weeks [0.000999 ***]). We also found that the upper GI (0.000999 ***) and the lower GI (0.000999 ***) were significantly different between 6 and 8 weeks of age. Additionally, while each sex varied between 6 and 8 weeks, the lower GI difference between female and male mice at 8 weeks (0.002997 **) was less significant than it was at 6 weeks (0.000999 ***). This was also supported by the beta dispersion plot in Figure 1B, where the 8-week timepoints seem to cluster closer together than the 6-week ones. We interpreted this to mean that the onset of sexual maturity is associated with a stabilized microbiome community in these mice. Furthermore, we clearly saw a distinct upper GI microbiome and a distinct lower GI microbiome within each timepoint, although the sex differences were not as evident (Figure 1B). Thus, as age increases, there is a change in the abundance of taxa between the upper and lower GI regions, and this trend is relatively consistent between male and female mice.

To better understand the sex-based variations in these GI regions, we looked at the taxonomic differences (genus) between males and females in the upper vs. lower GI, at age 6 and 8 weeks of age. In Figure 1C, we show the PSI and the colon as representatives for each region, but all relative abundance plots can be found in Supplementary Figure S1. Overall, the relative abundance in the lower GI samples demonstrated a greater variety of taxa than the upper GI. Yet, at 6 weeks of age, the relative abundance between males and females was visually similar in the colon. Meanwhile, in the PSI, males has an increase in *Muribaculaceae.* However, at 8 weeks of age, there were more obvious sex-differentiated blooms where females has more *Lactobacillus* than the males in the PSI, and males had more *Clostridium_sensu_stricto_1* (Figure 1C). Interestingly, female colons appeared to have more *Dubosiella* at 6 weeks, but this trend was reversed at 8 weeks, with male colons having higher *Dubosiella* abundance. The taxonomic differences in Figure 1C led us to conduct a statistical analysis utilizing DESeq to determine if these taxa were indeed differentially abundant (Figure 1D). In 6-week-old female mice, *Coriobacteriaceae_UCG-002* was consistently higher in both the PSI and colon (10 and 25-fold increase, respectively), compared to males. Meanwhile, in 6-week-old males, *Muribaculaceae* and *Clostridium_sensu_stricto_1* were higher in both the PSI and the colon (~30 log fold increase for all). Our *Dubosiella* finding was also supported with a 10-fold increase in female colons at 6 weeks and a 30-fold increase in male colons at 8 weeks. Overall, the most significantly increased taxa were entirely different at 8 weeks of age with no shared differentially abundant taxa between the PSI and colon in both sexes. We also found a greater number of differentially abundant taxa in the colon compared to the PSI, which was consistent with the relative abundance findings (Figure 1D). These results demonstrate that not only the sample’s GI origin is a main determinant in its differential abundance, but also the mice’s age and sex.

**Figure 2:**
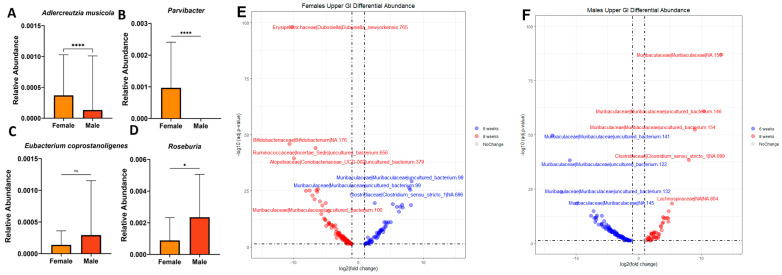
Taxa significantly associated with sex and age. (**A**–**D**) Taxa significantly associated with estradiol in females at 8 weeks of age identified using the MaAsLin2 package (v.1.4.0). Plotted relative abundance and Mann-Whitney statistical analysis. Significance values as follows: * *p* < 0.05 and **** *p* < 0.0001, ns = non-significant. (**E**) Female and (**F**) male upper GI at both 6 and 8 weeks old using the DESeq2 R package (v.1.40.2). Dashed lines indicate a p-adjusted value of 0.05.

We then measured hormonal serum concentrations, due to the potential significance of the mice reaching sexual maturity and their hypothesized importance in driving sex-based differences in the microbiome. We chose to measure three relevant hormones using ELISAs. Progesterone was chosen due to its prevalence in both sexes and its close relationship to growth [26]. We then chose more sex-specific hormones associated with sexual maturity in females and males—estradiol and testosterone, respectively. We conducted a two-way ANOVA on the raw hormone quantification values and found no significant differences between ages and sexes. However, we did observe significant dispersion within the samples. This may be due to various hormonal states in these animals. Therefore, we utilized MaAsLin2, a pipeline that can identify associations between multivariate metadata using linear modeling, to observe correlations between taxa and the three sex hormones measured in blood serum (Figure 2A–D). The strongest correlations were found with an estradiol concentration at 8 weeks of age.

MaAslin2 analysis found that the most significantly associated taxa with increasing estradiol at 8 weeks were *Adlercreutzia mucosicola*, *Eubacterium coprostanoligenes*, *Parvibacter*, and *Roseburia* (Figure 2A–D). Based on these Maaslin2 identified significances in relation to estradiol, we then wanted to see whether these taxa were also associated with sex differences at 8 weeks. *Adlercreutzia mucosicola* was found at a significantly higher relative abundance in females than in males (Figure 2A). *Adlercreutzia mucosicola* is a Gram-positive anaerobe reported to inhabit the intestinal mucosa in mice [27,28]. Interestingly, *Adlercreutzia mucosicola* is known to metabolize the phytoestrogens daidzein and genistein to equol, a metabolite with the highest affinity to estrogen receptors [29]. *Parvibacter* was found at significantly higher levels in female mice than in male mice (Figure 2B). It is worth noting that it belongs to the family *Eggerthellaceae*, and is phylogenetically similar to the *Alderceutzia* genus, suggesting that it may have a similar impact on estrogenic activity [30]. Its only known species is *Parvibacter caecicola*, and the metabolites it produces have not been well-elucidated [27]. However, our analysis was not able to resolve down to this level. *Eubacterium coprostanoligenes* was found at a higher relative abundance in males, but not significantly so (Figure 2C). *Eubacterium coprostanoligenes* reduces cholesterol to coprostanol, leading to lower cholesterol absorption and increased fecal coprostanol [31]. *Roseburia* was found in a significantly higher concentration in males (Figure 2D). Interestingly, it has been shown to have a positive correlation to blood testosterone levels in healthy males, and a negative correlation to low butyrate and butyrate-producing genes, and *Clostridium difficile* infection [32]. We used DESeq2 to determine if the notable differences between females and males at 8 weeks of age in the upper GI, identified with MaAsLin2, were replicated with a second methodology. DESeq2 indicated that *Adlercreutzia mucosicola* and *Parvibacter* are found at a −5.1 (*p* = 3.74 × 10^−10^) and −6.4 (*p* = 7.14 × 10^−14^) log2-fold change in the upper GI of 8-week-old male mice than in female mice, respectively (Supplementary Figure S2). Interestingly, we also found that *Adlercreutzia mucosicola* is significantly decreased in males at 6 weeks of age than at 8 weeks of age (Figure 2F).

We then wanted to determine whether any of the taxa significantly associated with sex hormones were differentially abundant in the upper GI at 6 weeks vs. 8 weeks, which is known as a pivotal location for nutrient uptake in the body. We utilized DESeq2 analysis to determine the taxa associated with female and male mice at both timepoints within a combination of all upper GI samples (Figure 2E,F). DESeq2 indicated that in 8-week-old females, *Dubosiella newyorkensis* is found at significantly lower quantities in the upper GI than it is at 6 weeks (Figure 2E). At 8 weeks of age, males have significantly more *Muribaculaceae*, *Lachnospiraceae*, and *Clostridia_sensu_stricto_1* than they do at 6 weeks (Figure 2F). *Muribaculaceae* is part of the “normal” flora of murine animals and contributes to the production of propionate in the gut [33]. *Lachnospiraceae* are an important family of bacteria contributing to butyrate production in the gut [34]. *Clostridia_sensu_stricto_1* has a negative correlation to the level of inflammatory markers found in the serum [35]. Overall, these findings are consistent with those observed in the hormonal analysis and the GI-region-specific DESeq2 results. This leads us to theorize that the sex-based differences we observed could, in part, be driven by hormonal levels, possibly within the GI environment.

## 4. Limitations and Conclusions

While our study has provided valuable insights into the interconnectivity of age, sex, and the gut microbiome in mice, it is essential to acknowledge its limitations. Our study primarily relied on 16S rRNA analysis, which offers taxonomic information, but lacks functional insights. Future research should consider employing metagenomic and metatranscriptomic approaches to better understand the functional significance of the observed microbial changes, as well as a better resolution of the taxonomic assignments at the species level. All experiments for this work were carried out with replicates spanning multiple experimental cohorts. Thus, the differences identified are likely more robust than ones observed from a single timepoint, as they would have to overcome this longitudinal variability in addition to cage effects. However, we cannot entirely rule out the presence of subtle cage effects that may contribute to the observed dispersion. Moreover, our age-related findings over a short two-week period highlight the need for more longitudinal studies that track microbiome changes over an extended period to ascertain whether the observed trends persist or evolve over time. We employed a cohort comprising 32 mice (distributed evenly into 8 mice per experimental group), but we acknowledge that more mice may be required to identify rare events and changes in lower abundance taxa. Additionally, while ELISA analyses have been shown to be reliable in several works [36,37,38,39], we observed a large dispersion within our own samples. Some of the variability could be due to not taking into account the estrous cycle in the female mice. Regardless, we believe that a follow-up work using mass spectrometry to determine hormonal concentrations would be prudent. Finally, as is the limitation in many microbiome studies, the translatability of our results is limited by the mice all being sourced from the same vendor. Repeating this work with data from several vendors would help mitigate this.

In summary, this work demonstrated that there are significant taxonomic differences between male and female mice, which are heavily influenced by both gut geography and age. We observed notable taxonomic differences in the upper gastrointestinal tract that were influenced by both age and sex. Specifically, the proximal small intestine exhibited a significant shift in microbial diversity as the mice reached sexual maturity, emphasizing the importance of age in shaping the gut microbiota. Furthermore, our investigation revealed associations between specific taxa and sex hormones, shedding light on potential mechanisms underlying sex-related differences in the murine microbiome. These findings underscore the complexity of microbiome dynamics and its responsiveness to hormonal changes. Future works should ensure to take all of these factors into account to ensure that their results are truly representative and relevant to potential health implications.

## Data Availability

As of the date of publication, the data from this study are publicly available in the NCBI Short Read Archive (SRA) under BioProject ID number PRJNA1064926.

## Supplementary Materials

The following supporting information can be downloaded at: https://www.mdpi.com/article/10.3390/microorganisms12010221/s1, Figure S1: Relative abundance plots per GI region for female and male mice at 6 and 8 weeks old. Figure S2: Differential abundance DESeq2 plot between female and male mice in the upper GI at 8 weeks old.

## Abbreviations

GI	Gastrointestinal tract
ELISA	Enzyme-linked immunosorbent assay
PSI	Proximal small intestine
MSI	Middle small intestine
DSI	Distal small intestine
