# Supplementary material for: Gut Biogeography Accentuates Sex-Related Differences in the Murine Microbiome
Source: Microorganisms. 2024 Jan 22;12(1):221. doi: 10.3390/microorganisms12010221 (PMC10821414; doi:10.3390/microorganisms12010221)
Supplement: Supplementary file 1 [file microorganisms-12-00221-s001.zip › microorganisms-2798201-supplementary.pdf]

Supplementary Figures

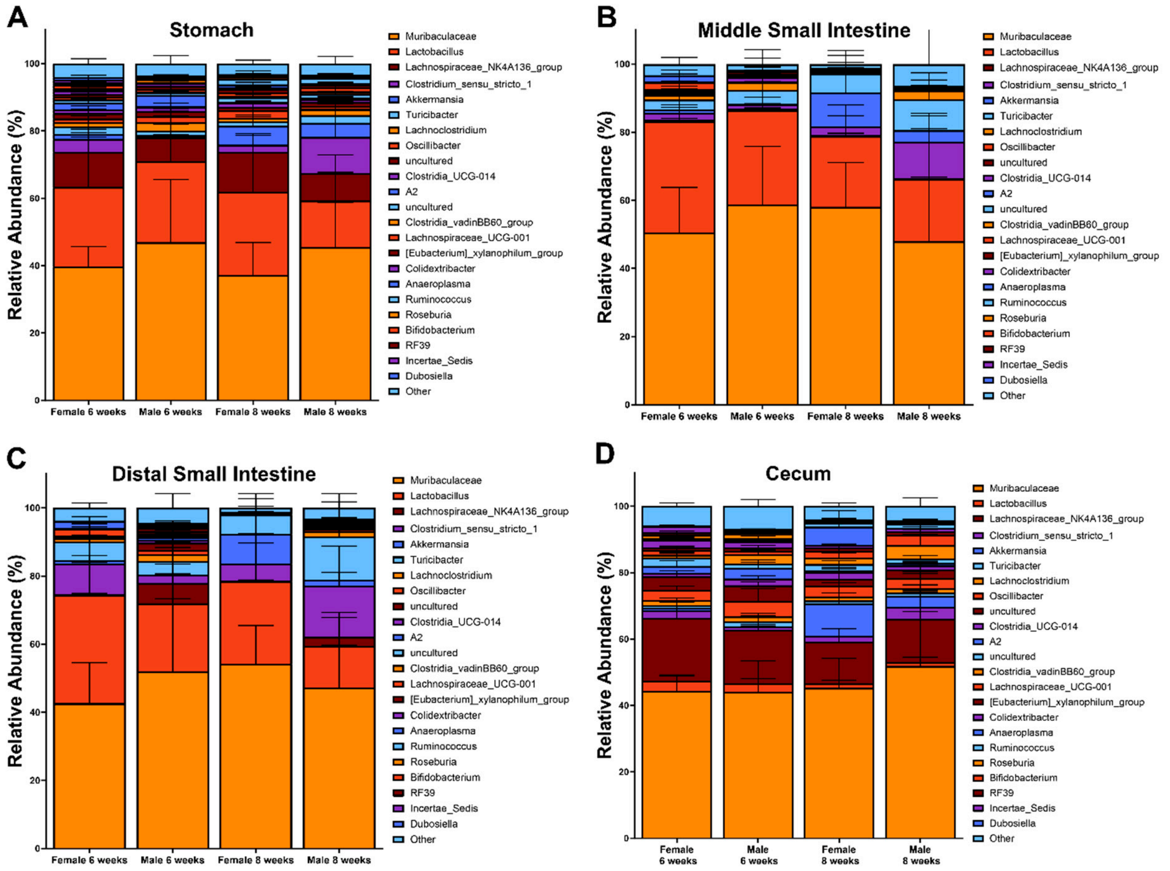

Supplementary Figure S1: Relative abundance plots per GI region for female and male mice at 6 and 8 weeks of age. A) Stomach, B) middle small intestine, C) distal small intestine, and D) cecum.

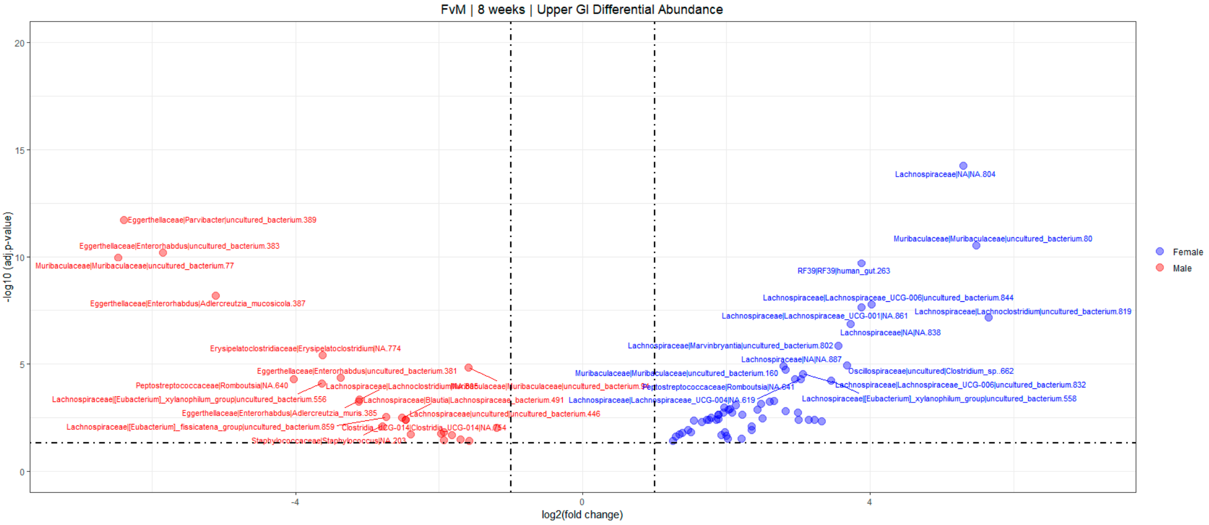

Supplementary Figure S2: Differential abundance DESeq2 plot between female and male mice in the upper GI at 8 weeks of age.
